# Supplementary material for: A novel epigenetic AML1‐ETO/THAP10/miR‐383 mini‐circuitry contributes to t(8;21) leukaemogenesis
Source: EMBO Mol Med. 2017 May 24;9(7):933–49. doi: 10.15252/emmm.201607180 (PMC5577530; doi:10.15252/emmm.201607180)
Supplement: Supplementary file 1 — Appendix [file EMMM-9-933-s001.pdf]

EMM-2016-07180_A novel epigenetic *AML1-ETO/THAP10/miR-383* mini-circuitry contributes to leukemogenesis

Appendix

Inventory of Appendix Information

Appendix Data

Appendix Table S1

Appendix Table S2

Appendix Table S3

Appendix Table S4

Appendix Table S5

Appendix Table S6

Appendix Table S7

Appendix Figure S1

Appendix Figure S2

Appendix Figure S3

Appendix Figure S4

Appendix Figure S5

Appendix References

Appendix Data

Appendix Table S1. Morphological and genetic features of primary AML samples (n = 40) for DNA methylation profiling

Pt #	t(8;21)	Morphology by FAB*	Genotype	Karyotype
1	+	AML/M2	*AML1/ETO*	45,X,t(8;21)(q22;q22)+mar, 11q+,-Y[2]/46,XY,t(8;21)(q22;q22)[9]/46,XY[9]
2	+	AML/M2	*AML1/ETO*	46,XY,t(8;21)(q22;q22)[10]/45,X,-Y[10]
3	+	AML/M2	*AML1/ETO*	45,X,-Y,t(8;21)(q22;q22)[20]
4	+	AML/M2	*AML1/ETO*	46,XY,t(8;21)(q22;q22)[4]/45,idem,-Y[8]/46,XY[8]
5	+	AML/M2	*AML1/ETO*	45,X,-Y,t(8;21)(q22;q22)[20]
6	+	AML/M2	*AML1/ETO*	46,XY,t(8;21)(q22;q22)[8]/46,XY[12]
7	+	AML/M2	*AML1/ETO*	46,XX,t(8;21)(q22;q22)[20]
8	+	AML/M2	*AML1/ETO*	46,XX,t(2;8;21)(q37;q22;q22)[8]/46,XX,t(8;21)(q22;q22)[6]/ 46,XY[6]
9	+	AML/M2	*AML1/ETO*	46,XY,t(8;21)(q22;q22)[20]
10	+	AML/M2	*AML1/ETO*	46,XY,t(8;12;21)(q22;q13;q22)[10]/46,XY,t(8;21)(q22;q22)[8]/46,XY[2]
11	+	AML/M2	*AML1/ETO*	46,XX,t(8;21)(q22;q22)[15]/46,XX,t(8;21)(q22;q22),del(9)(q 22)[5]
12	+	AML/M2	*AML1/ETO*	46,XX,-4,+8,t(8;21)(q22;q22)[20]
13	−	AML/M2	*FLT3-ITD* *NPM1* mut	45,XY,-3,+8,+12,-17 [4]/46,XY[16]
14	−	AML/M2	*NPM1* mut	46,XX [20]
15	−	AML/M2	*AML1/MTG16*	46,XY,t(16;21)(q24;q22),+8,-17 [6]/46,XY[14]
16	−	AML/M2	*FLT3* OE	46,XY [20]
17	−	AML/M2	*NRAS* mut	46,XY [20]
18	−	AML/M2	*MLL/AF9*	46,XY,t(9;11)(p21;q23)[10]/46,XY,del(9)(q21)[8]/46,XY[2]
19	−	AML/M2	*AML1/MDS1/ EVI1*	46,XY [20]
20	−	AML/M2	*AML1/MTG16*	46,XY,t(16;21)(q24;q22),-1,+8[18]/46,XY[2]
21	−	AML/M2	*MLL/ELL*	44,XY,t(11;19)(p21;q23),-3,-5[10]/ 46,XY[10]
22	−	AML/M2	*MLL* mut	46,XX [20]
23	−	AML/M2	*MLL/ELL*	46,XY,t(11;19)(p21;q23)[2]/46,XY [18]
24	−	AML/M2	*FLT3-ITD*	46,XX [20]
25	−	AML/M2	*CBFB/MYH11*	46,XY [20]
26	−	AML/M2	*MLL/AF9*	46,XY,t(9;11)(p21;q23)[6]/46,XY,del(17)(q22)[8]/46,XY[6]
27	−	AML/M2	*WT1* OE	46,XY,+8,-17 [8]/46,XY[12]
28	−	AML/M2	*NPM1* mut	46,XX [20]
29	−	AML/M2	*CEBPA* mut	46,XX [20]
30	−	AML/M2	*KIT OE*	46,XY [20]
31	−	AML/M2	*HOXA9* OE	46,XY [20]
32	−	AML/M2	*AML1/MDS1*	47,XX,+22 [2]/46,XY[18]
33	−	AML/M2	*NPM1* mut	46,XY [20]
34	−	AML/M2	*NRAS* mut	46,XY [20]
35	−	AML/M2	*MLL/MLL*	47,XY,+11 [2]/46,XY[18]
36	−	AML/M2	*TLS/ERG*	46,XX,t(16;21)(p11;q22) [9]/46,XX,1q+[11]
37	−	AML/M2	*MLL/AF6*	46,XX [20]
38	−	AML/M2	*NRAS* mut	46,XY [20]
39	−	AML/M2	*FLT3-ITD,* *NPM1* mut	45,XY,-3,+8,+12,-17 [4]/46,XY[16]
40	−	AML/M2	*NPM1* mut	46,XX [20]

∗Diseases are classified according to the FAB classification (Bennett et al, 1985).The number of mitotic cells acquired is indicated in []. Mut, mutation; OE, over-expression.

Appendix Table S2. Pathway analysis of hypermethylated genes in the AML1-ETO^{+} AML DNA methylation signature

Pathway	Count	P value	Gene list
Pathways in cancer	57	5.50E-04	FYN RASGRF1 ARHGAP5 SHC1 SHC2 ACTB CAPN2 COMP CAV1 CAV2 COL1A1 COL5A1 COL6A1 COL11A1 COL11A2 CCND1 CCND2 DOCK1 DIAPH1 FLT4 GSK3B IGF1R ITGA11 ITGA2 ITGA9 ITGB7 LAMA5 LAMB2 MAP2K1 MYL10 MYL5 PAK7 PIP5K1C PIK3CD PIK3R2 PIK3R5 PGF PDGFA PDGFB TNXB THBS1 THBS2 AKT1 ERBB2 SRC VEGFC VAV2
Focal adhesion	56	3.00E-05	BCL2L1 CTBP2 CREBBP E2F1 GLI1 RASSF5 TRAF1 AXIN1 BMP4 CASP9 CCNA1 CCND1 EGLN1 EGLN2 EGLN3 FGF14 FGF17 FGF18 FGFR3 FOXO1 FZD1 FZD2 FZD5 FZD9 GSK3B HHIP IGF1R ITGA2 LAMA5 LAMB2 MAP2K1 NTRK1 PTCH1 PIK3CD PIK3R2 PIK3R5 PLCG1 PGF PDGFA PDGFB RET RB1 RARA RARB RXRA RUNX1T1 KIT SHH TCF7L2 AKT1 ERBB2 VEGFC WNT10B WNT11 WNT5B WNT7A
Wnt signaling pathway	16	8.00E-03	GLI1 AXIN1 BMP4 FZD1 FZD2 FZD5 FZD9 GSK3B HHIP PTCH1 SHH TCF7L2 WNT10B WNT11 WNT5B WNT7A
Cell adhesion molecules (CAMs)	29	3.30E-02	CTBP2 CREBBP WIF1 AXIN1 CHP2 CAMK2D CSNK1E CUL1 CCND1 CCND2 DKK1 FRAT1 FZD1 FZD2 FZD5 FZD9 GSK3B LRP5 CSNK2B NFATC1 PLCB2 PRKX PPP3CA TCF7L2 VANGL2 WNT10B WNT11 WNT5B WNT7A
Apoptosis	12	2.70E-03	CCNA1 CCND1 MAP2K1 PIK3CD PIK3R2 PIK3R5 RARA RPS6KB2 RUNX1T1 KIT TCF7L2 AKT1
Basal cell carcinoma	12	7.00E-04	CCNA1 CCND1 MAP2K1 PIK3CD PIK3R2 PIK3R5 RARA RPS6KB2 RUNX1T1 KIT TCF7L2 AKT1
Chronic myeloid leukemia	16	1.70E-02	
Hematopoietic cell lineage	23	9.20E-02	CD226 CD40 CDH15 CDH5 CLDN15 CNTN1 ESAM ITGA9 ITGAL ITGB2 ITGB7 HLA-E HLA-DMA HLA-DMB HLA-DPA1 HLA-DRA MADCAM1 NRXN2 PVRL1 PDCD1 SELL SDC3 VCAN
ErbB signaling pathway	16	9.90E-02	CD14 CD19 CD3E CD5 CD7 CD9 FCER2 DNTT GP9 ITGA2 IL11 IL2RA IL7 HLA-DRA KIT TPO
Acute myeloid leukemia	17	9.80E-03	NCK2 SHC1 SHC2 BTC CAMK2D GSK3B MAP2K1 NRG4 PAK7 PIK3CD PIK3R2 PIK3R5 PLCG1 RPS6KB2 AKT1 ERBB2 SRC

Appendix Table S3. Top 10 genes which methylation levels are higher in AML1-ETO^{+} than AML1-ETO^{-}blasts and normal bone marrow cells

WikiGene Name	Chromosome	P value	WikiGene Description
C15orf2	Chr15	0	chromosome 15 open reading frame 2
LRRC49	Chr15	0	leucine rich repeat containing 49
THAP10	Chr15	0	THAP domain containing 10
CDH18	Chr5	1E-6	cadherin 18, type 2
GPR45	Chr2	1E-6	G protein-coupled receptor 45
MINPP1	Chr10	2E-6	multiple inositol-polyphosphate phosphatase 1
SEPT9	Chr17	1E-6	septin 9
DKK1	Chr10	2E-6	dickkopf homolog 1 (Xenopuslaevis)
PREX2	Chr8	3E-6	phosphatidylinositol-3,4,5-trisphosphate-dependent Rac exchange factor 2
TUBA3C	Chr13	4E-6	tubulin, alpha 3c

Appendix Table S4. Morphological and genetic features of primary AML samples (n = 28) for analysis of *THAP10* expression

Pt#	Morphology by FAB*	Genotype	Karyotype
1	AML/M0	*DEK/CAN*	46,XY,t(6;9)(p23;q34)[10]/46,XY[10]
2	AML/M0	*MLL/ELL*	46,XY,t(11;19)(p21;q23)[9]/45,XY,-20[11]
3	AML/M0	*DEK/CAN*	46,XY [20]
4	AML/M1	*SET/CAN*	46,XX,-7, +22[6]/45,XX,-7[10]/46,XX[14]
5	AML/M1	*AML1/MDS1*	45,XY,-5, -20, +8[9]/45,XY,-5[7]/46,XY[4]
6	AML/M1	*RUNX1* mut	46,XX,-5, -20, +8,+22 [20]
7	AML/M2	*AML1/ETO, KIT* mut	46,XY,t(8;21)(q22;q22)[20]
8	AML/M2	*AML1/ETO, KIT* mut	46,XX,t(8;21)(q22;q22)[16]/46,XX,10p-[1]/46,XX[3]
9	AML/M2	*AML1/ETO, KIT* mut	46,XY,t(8;21)(q22;q22),-21,+22[10]/46,XY,t(8;21)(q22;q22)[4]/46,XY[6]
10	AML/M2	*AML1/MTG16*	46,XY,t(16;21)(q24;q22)[15]/46,XY[5]
11	AML/M2	*MLL/ELL, HOXA9* OE	46,XY,t(11;19)(p21;q23)[8]/45,XY,del(20)(q11)[12]
12	AML/M2	*MLL/AF9, HOXA9* OE	46,XY,t(9;11)(p21;q23)[12]/46,XY,del(17)(q22)[8]
13	AML/M3	*PML/RARα, FLT-ITD*	46,XX,t(15;17)(q22;q21)[10]/46,XX[10]
14	AML/M3	*PML/RARα*	46,XY,t(15;17)(q22;q21)[18]/46,XY,+8,-1[2]
15	AML/M3	*PML/RARα*	46,XY,t(15;17)(q22;q21)[8]/ 46,XY,del(7)(q22)[12]
16	AML/M4	*NUP98/HOXA9*	46,XX,t(7;11)(p15;p15)[4]/46,XX[16]
17	AML/M4	*NRAS* mut, *HOXA9* OE	46,XX ,i(12)(q10)[6]/ 46,XX[14]
18	AML/M4	*AML1/MTG16*	46,XY [20]
19	AML/M4eo	*CBFβ/MYH11*	46,XY [20]
20	AML/M4eo	*CBFβ/MYH11*	46,XX [20]
21	AML/M4eo	*CBFβ/MYH11*	46,XY [20]
22	AML/M5	*MLL/AF10*	46,XX [20]
23	AML/M5	*MLL/AF6*	46,XY,t(6;11)(q27;q23)[12]/46,XY [8]
24	AML/M5	*TLS/ERG*	46,XY,t(16;21)(p11;q22)[17]/46,XY[3]
25	AML/M6	*NRAS* mut	46,XX, 22q+ [10]/46,XX[10]
26	AML/M6	*HOXA9* OE	46,XY [20]
27	AML/M7	*MLL/PTD*	46,XX [20]
28	AML/M7	*MLL/PTD*	46,XX [20]

*Diseases are classified according to the FAB classification (Bennett et al, 1985). The number of mitotic cells acquired is indicated in []. Mut, mutation; OE, over-expression.

Appendix Table S5. Morphological and genetic features of primary AML samples (n= 124) for validation of *THAP10* expression

Pt #	t(8;21)	Morphology by FAB*	Genotype	Karyotype
1	−	AML/M2	*MOZ/CBP,FLT3-TKD*	46,XX [20]
2	−	AML/M2	*AML1/MTG16*	46,XY,t(16;21)(q24;q22)[15]/46,XY[5]
3	−	AML/M2	*MLL/ELL*	46,XY,t(11;19)(p21;q23)[8]/46,XY,del(20)(q11)[12]
4	−	AML/M2	*MLL/AF9*	46,XY,t(9;11)(p21;q23)[12]/46,XY,del(17)(q22)[8]
5	−	AML/M2	*AML1/EVI1*	46,XX [20]
6	−	AML/M2	*NPM1* mutation	46,XY [20]
7	−	AML/M2	*BAALC* OE	46,XY [20]
8	−	AML/M2	*GATA2* mut	46,XY [20]
9	−	AML/M2	*TEL/PDGFRB*	46,XX [20]
10	−	AML/M2	*GATA2*OE	46,XX [20]
11	−	AML/M2	*MLL/AF9*	46,XY,t(9;11)(p21;q23)[10]/46,XY,del(9)(q21)[8]/46,XY[2]
12	−	AML/M2	*AML1/MDS1/EVI1*	46,XY [20]
13	−	AML/M2	*AML1/MTG16*	46,XY,t(16;21)(q24;q22),-1,+8[18]/46,XY[2]
14	−	AML/M2	*MLL/ELL*	44,XY,t(11;19)(p21;q23),-3,-5[10]/ 46,XY[10]
15	−	AML/M2	*MLL* mut	46,XX [20]
16	−	AML/M2	*MLL/ELL*	46,XY,t(11;19)(p21;q23)[2]/46,XY [18]
17	−	AML/M2	*FLT3-ITD*	46,XX [20]
18	−	AML/M2	*CBFB/MYH11*	46,XY [20]
19	−	AML/M2	*MLL/AF9*	46,XY,t(9;11)(p21;q23)[6]/46,XY,del(17)(q22)[8]/46,XY[6]
20	−	AML/M2	*WT1* OE	46,XY,+8,-17 [8]/46,XY[12]
21	−	AML/M2	*NPM1* mut	46,XX [20]
22	−	AML/M2	*CEBPA*mut	46,XX [20]
23	−	AML/M2	*KIT* OE	46,XY [20]
24	−	AML/M2	*HOXA9* OE	46,XY [20]
25	−	AML/M2	*AML1/MDS1*	47,XX,+22 [2]/46,XY[18]
26	−	AML/M2	*NPM1* mut	46,XY [20]
27	−	AML/M2	*NRAS* mut	46,XY [20]
28	−	AML/M2	*MLL/MLL*	47,XY,+11 [2]/46,XY[18]
29	−	AML/M2	*TLS/ERG*	46,XX,t(16;21)(p11;q22) [9]/46,XX,1q+[11]
30	−	AML/M2	*MLL/AF6*	46,XX [20]
31	−	AML/M2	*NRAS* mut	46,XY [20]
32	−	AML/M2	*FLT3-ITD,NPM1* mut	45,XY,-3,+8,+12,-17 [4]/46,XY[16]
33	−	AML/M2	*NPM1* mut	46,XX [20]
34	−	AML/M2	*FLT3* OE	46,XY [20]
35	−	AML/M2	*MLL/ELL*	46,XY,t(11;19)(p21;q23)[3]/46,XY [17]
36	−	AML/M2	*AML1/MTG16*	46,XY,t(16;21)(q24;q22),+8, -17 [6]/46,XY[14]
37	−	AML/M2	*GATA2* OE	46,XX [20]
38	−	AML/M2	*CEBPA* mut	46,XX [20]
39	−	AML/M2	*GATA1* OE	46,XY [20]
40	−	AML/M2	*FLT3* OE	46,XY [20]
41	−	AML/M2	*NRAS* mut	46,XY [20]
42	−	AML/M2	*AML1/MTG16*	46,XY,t(16;21)(q24;q22),-1,+8[16]/46,XY[4]
43	−	AML/M2	*MLL/ELL*	44,XY,t(11;19)(p21;q23),-3,-5[8]/ 46,XY[12]
44	−	AML/M2	*MLL* mut	46,XX [20]
45	−	AML/M2	*MLL/ELL*	46,XY,t(11;19)(p21;q23)[4]/46,XY [16]
46	−	AML/M2	*FLT3-ITD*	46,XX [20]
47	−	AML/M2	*CBFB/MYH11*	46,XY [20]
48	−	AML/M2	*TLS/ERG*	46,XX,t(16;21)(p11;q22) [10]/46,XX,1q+[10]
49	+	AML/M2	*AML1/ETO,KIT* mut	46,XY,t(8;21)(q22;q22)[20]
50	+	AML/M2	*AML1/ETO,KIT* mut	46,XX,t(8;21)(q22;q22)[16]/46,XX,10p[1]/46,XX[3]
51	+	AML/M2	*AML1/ETO,KIT* mut	46,XY,t(8;21)(q22;q22),-21,+22[10]/46,XY,t(8;21)(q22;q22)[4] /46,XY[6]
52	+	AML/M2	*AML1/ETO*	46,XY,t(8;21)(q22;q22)+mar, 22q-[20]
53	+	AML/M2	*AML1/ETO,KIT* mut	46,XY,t(8;21)(q22;q22)[20]

54	+	AML/M2	*AML1/ETO*,*KIT* mut	45,X,t(8;21)(q22;q22),-Y[9]/ 46,XY,t(8;21)(q22;q22)[11]
55	+	AML/M2	*AML1/ETO*	46,XY,t(8;21)(q22;q22)[7]/ 45,X, t(8;21)(q22;q22),-Y [13]
56	+	AML/M2	*AML1/ETO*	47,XY,t(8;21)(q22;q22),+22[10]/46,XY,t(8;21)(q22;q22)[6] /46,XY[4]
57	+	AML/M2	*AML1/ETO*	46,XX,t(8;21)(q22;q22)[20]
58	+	AML/M2	*AML1/ETO*,*KIT* mut	46,X,t(8;21)(q22;q22),-Y,+8[16]/46,XY,t(8;21)(q22;q22)[4]
59	+	AML/M2	*AML1/ETO*	46,XY,t(8;21)(q22;q22)+mar,11q+[20]
60	+	AML/M2	*AML1/ETO*	46,XY,t(8;21)(q22;q22)[20]
61	+	AML/M2	*AML1/ETO*	46,XX,t(8;21)(q22;q22)[20]
62	+	AML/M2	*AML1/ETO*	46,XY,t(8;21)(q22;q22)[20]
63	+	AML/M2	*AML1/ETO*,*KIT* mut	46,X,t(8;21)(q22;q22),+22,+8,-17,-Y[6]/46,XY,t(8;21)(q22;q22),7p-[2]/46,XY[12]
64	+	AML/M2	*AML1/ETO*	45,XX,t(8;21)(q22;q22), -X[10]/46, XX[10]
65	+	AML/M2	*AML1/ETO*	46,XX,t(8;21)(q22;q22)[6]/46,XX,7q-[4]/46,XX[10]
66	+	AML/M2	*AML1/ETO*	46,XY,t(8;21)(q22;q22),-5,+17[2]/46,XY,t(8;21)(q22;q22)[6] /46,XY[12]
67	+	AML/M2	*AML1/ETO*	46,XY,t(8;21)(q22;q22)+mar, 5q-[9]/46,XY[11]
68	+	AML/M2	*AML1/ETO*	46,XX,t(8;21)(q22;q22)[20]
69	+	AML/M2	*AML1/ETO*,*KIT* mut	45,X,t(8;21)(q22;q22),-Y[2]/46,XY,t(8;21)(q22;q22)[18]
70	+	AML/M2	*AML1/ETO*	46,XY,t(8;21)(q22;q22)[7]/ 46,XY[13]
71	+	AML/M2	*AML1/ETO*	45,XY,t(8;21)(q22;q22),-5,+8,-17,+22, [3]/ 46,XY[17]
72	+	AML/M2	*AML1/ETO*	46,XX,t(8;21)(q22;q22)[20]
73	+	AML/M2	*AML1/ETO*	45,X,t(8;21)(q22;q22),-Y[16]/ 46,XY [4]
74	+	AML/M2	*AML1/ETO*	46,XY,t(8;21)(q22;q22)+mar, 1q+[8]/46,XY[12]
75	+	AML/M2	*AML1/ETO*	46,XY,t(8;21)(q22;q22)[20]
76	+	AML/M2	*AML1/ETO*	46,XX,t(8;21)(q22;q22)[20]
77	+	AML/M2	*AML1/ETO*	44,X,t(8;21)(q22;q22),-3,-Y[11]/46,XY[9]
78	+	AML/M2	*AML1/ETO*	45,X,t(8;21)(q22;q22),-X[10]/46,XX,t(8;21)(q22;q22)[10]
79	+	AML/M2	*AML1/ETO*	47,XY,t(8;21)(q22;q22)+mar,+11 [5]/46,XX [15]
80	+	AML/M2	*AML1/ETO*	46,XY,t(8;21)(q22;q22)[7]/46,XY[13]
81	+	AML/M2	*AML1/ETO*	46,XX,t(8;21)(q22;q22)[20]
82	+	AML/M2	*AML1/ETO*	46,XY,t(8;21)(q22;q22)[20]
83	+	AML/M2	*AML1/ETO*	45,X,t(8;21)(q22;q22),-Y[16]/ 46,XY,t(8;21)(q22;q22)[4]
84	+	AML/M2	*AML1/ETO*	45,X,t(8;21)(q22;q22)+mar,11q+,-85Y[2]/46,XY,t(8;21)(q22;q22)[9]/46,XY[9]
85	+	AML/M2	*AML1/ETO*	4686,XY,t(8;21)(q22;q22)[8]/46,XY[12]
86	+	AML/M2	*AML1/ETO*	46,XX,t(8;21)(q22;q22)[20]
87	+	AML/M2	*AML1/ETO*	47,XY,t(8;21)(q22;q22),+13, -19,+21 [7]/46,XY[13]
88	+	AML/M2	*AML1/ETO*	46,XY,t(8;21)(q22;q22)[20]
89	+	AML/M2	*AML1/ETO*,*KIT* mut	46,XY,t(8;21)(q22;q22)[20]
90	+	AML/M2	*AML1/ETO*,*KIT* mut	46,XX,t(8;21)(q22;q22)[15]/46,XX,10p-[2]/46,XX[3]
91	+	AML/M2	*AML1/ETO*,*KIT* mut	46,XY,t(8;21)(q22;q22),-21,+22[12]/46,XY,t(8;21)(q22;q22)[4] /46,XY[4]
92	+	AML/M2	*AML1/ETO*	46,XY,t(8;21)(q22;q22)+mar, 22q-[20]
93	+	AML/M2	*AML1/ETO*,*KIT* mut	46,XY,t(8;21)(q22;q22)[20]
94	+	AML/M2	*AML1/ETO*,*KIT* mut	45,X,t(8;21)(q22;q22),-Y[10]/46,XY,t(8;21)(q22;q22)[10]
95	+	AML/M2	*AML1/ETO*	46,XY,t(8;21)(q22;q22)[8]/ 45,X, t(8;21)(q22;q22),-Y [12]
96	+	AML/M2	*AML1/ETO*	47,XY,t(8;21)(q22;q22),+22[10]/46,XY,t(8;21)(q22;q22)[4] /46,XY[6]
97	+	AML/M2	*AML1/ETO*	46,XX,t(8;21)(q22;q22)[20]
98	+	AML/M2	*AML1/ETO*,*KIT* mut	46,X,t(8;21)(q22;q22),-Y,+8[14]/46,XY,t(8;21)(q22;q22)[6]
99	+	AML/M2	*AML1/ETO*	46,XY,t(8;21)(q22;q22)+mar, 11q+[20]
100	+	AML/M2	*AML1/ETO*	46,XY,t(8;21)(q22;q22)[20]

101	+	AML/M2	*AML1/ETO*	46,XX,t(8;21)(q22;q22)[20]
102	+	AML/M2	*AML1/ETO*	46,XY,t(8;21)(q22;q22)[20]
103	+	AML/M2	*AML1/ETO*,*KIT* mut	46,X,t(8;21)(q22;q22),+22,+8,-17,-Y[4]/46,XY,t(8;21)(q22;q22),7p-[4]/46,XY[12]
104	+	AML/M2	*AML1/ETO*	45,XX,t(8;21)(q22;q22), -X[10]/46, XX[10]
105	+	AML/M2	*AML1/ETO*	46,XX,t(8;21)(q22;q22)[6]/46,XX,7q-[4]/46,XX[10]
106	+	AML/M2	*AML1/ETO*	46,XY,t(8;21)(q22;q22),-5,+17[4]/46,XY,t(8;21)(q22;q22)[4] /46,XY[12]
107	+	AML/M2	*AML1/ETO*	46,XY,t(8;21)(q22;q22)+mar, 5q-[8]/46,XY[12]
108	+	AML/M2	*AML1/ETO*	46,XX,t(8;21)(q22;q22)[20]
109	+	AML/M2	*AML1/ETO*,*KIT* mut	45,X,t(8;21)(q22;q22),-Y[3]/46,XY,t(8;21)(q22;q22)[17]
110	+	AML/M2	*AML1/ETO*	46,XY,t(8;21)(q22;q22)[7]/ 46,XY[13]
111	+	AML/M2	*AML1/ETO*	45,XY,t(8;21)(q22;q22),-5,+8,-17,+22, [4]/ 46,XY[16]
112	+	AML/M2	*AML1/ETO*	46,XX,t(8;21)(q22;q22)[20]
113	+	AML/M2	*AML1/ETO*	45,X,t(8;21)(q22;q22),-Y[14]/ 46,XY [6]
114	+	AML/M2	*AML1/ETO*	46,XY,t(8;21)(q22;q22)+mar, 1q+[8]/46,XY[12]
115	+	AML/M2	*AML1/ETO*	46,XY,t(8;21)(q22;q22)[20]
116	+	AML/M2	*AML1/ETO*	46,XX,t(8;21)(q22;q22)[20]
117	+	AML/M2	*AML1/ETO*	44,X,t(8;21)(q22;q22),-3,-Y[10]/46,XY[10]
118	+	AML/M2	*AML1/ETO*	46,XY,t(8;21)(q22;q22)[20]
119	+	AML/M2	*AML1/ETO*,*KIT* mut	46,XY,t(8;21)(q22;q22)[20]
120	+	AML/M2	*AML1/ETO*,*KIT* mut	46,XX,t(8;21)(q22;q22)[12]/46,XX,10p-[2]/46,XX[8]
121	+	AML/M2	*AML1/ETO*,*KIT* mut	46,XY,t(8;21)(q22;q22),-21,+22[10]/46,XY,t(8;21)(q22;q22)[4] /46,XY[6]
122	+	AML/M2	*AML1/ETO*	46,XY,t(8;21)(q22;q22)+mar, 22q-[20]
123	+	AML/M2	*AML1/ETO*,*KIT* mut	46,XY,t(8;21)(q22;q22)[20]
124	+	AML/M2	*AML1/ETO*	47,XY,t(8;21)(q22;q22),+22[12]/46,XY,t(8;21)(q22;q22)[4] /46,XY[4]

∗Diseases are classified according to the FAB classification (Bennett et al, 1985). The number of mitotic cells acquired is indicated in []. Mut, mutation; OE, over-expression.

Appendix Table S6. GO analysis of down-regulated genes in Kasumi-1 cells transduced with lenti-*THAP10*

GO-Term	Count	Gene list
Enzyme linked receptor protein signaling pathway	29	TWSG1, FGFR3, PDGFA, PML, ABI1, KIT, PTEN, EPHB2, IGF1R, EIF4EBP2, PDGFC, ANGPT1, INSR, GNG7, TXNIP, PTPRE,STAP1, PTPRG, FLT3, SMAD6, SMAD3,TRIO, HGF, RPS6KA5, EPS15, SS18, DOK4, SMURF2, JAK2
Protein kinase cascade	30	FGFR3, ZAK, ADORA2B, C5, PRKAG2, DUSP10, NFKBIA, KIT, CD74, BTK, IGF1R,RASGRP3, CHRNA7, CHRFAM7A, INSR,PTPRC,CCM2,TNIK,TIFA,MALT1,MAPK11,HGF,SHANK3, DAPK1,RPS6KA5,MAP4K4,SS18,RPS6KA4,DOK4,JAK2,DUSP9
Transmembrane receptor protein tyrosine kinase signaling pathway	21	TXNIP, FGFR3, STAP1, PTPRG, FLT3, PDGFA, ABI1, KIT, HGF, PTEN, EPHB2, RPS6KA5,EPS15, SS18, IGF1R, EIF4EBP2, DOK4,JAK2, ANGPT1, PDGFC, INSR
Negative regulation of cell differentiation	20	TWSG1, TBX3, FLT3, DLL3, PAX6, SMAD3, NFKBIA, CDK6, KIT, ZBTB16, GLI3, TTC3, CD74, EPHB2, NOTCH3, DLX2, MIB1, DLX1,RUNX1, ZFHX3
Regulation of programmed cell death	52	MEF2C, ZAK, ITSN1, PTEN, GLI3, BTK, CASP4, BAG1,CD44,RPS3A,CASP8,NMNAT3, BCL2,IFI16, BCL2L13, DAPK1, UNC13D, CSTB, TNFAIP3, PAFAH2, APH1B, CLU,PML, NFKBIA, SOX4, KIT, ZBTB16, ITM2B, CD74, GCH1, PLAGL1, IGF1R,TRAF5, PHLDA1,TRAF3,TXNIP,PTPRC,VAV3,TBX3,SMAD6,PDCD6,TRIO,MALT1,GAS1,HGF,CARD6,PPIF,DLX1,CDKN1A,SMAD3,JAK2,BIK
Hemopoiesis	21	PTPRC,TWSG1,LYN,FLT3,RELB,PML,KITLG,SOX4,CDK6,MALT1,KIT,ITGA4,IFI16,ZBTB16,CD74,TAL1,BCL2,CASP8,JAK2,RUNX1,HDAC9
Regulation of cell death	52	MEF2C,ZAK,ITSN1,PTEN,GLI3,BTK,CASP4,BAG1,CD44,RPS3A,CASP8,NMNAT3,BCL2,IFI16,BCL2L13,DAPK1,UNC13D,CSTB,TNFAIP3,PAFAH2,APH1B,CLU,PML,NFKBIA,SOX4,KIT,ZBTB16,ITM2B,CD74,GCH1,PLAGL1,IGF1R,TRAF5,PHLDA1,TRAF3,TXNIP,PTPRC,VAV3,TBX3,SMAD6,JAK2,BIK,PDCD6, SMAD3,TRIO,MALT1,GAS1,HGF,CARD6,PPIF,DLX1, CDKN1A
Intracellular signaling cascade	72	NFATC1,PDK1,PTPRC,CCM2,VAV3,RABIF,ARFIP2,MAPK11,MALT1,NPR3,HGF,SHANK3,RAB33B,TRAF3IP2,RPS6KA5,P2RY11,RPS6KA4,RAP1B,JAK2, ZAK,PRKAG2,IQGAP2,CXCR2,ITSN1,STAC3,BTK,MCTP2,EIF4EBP2,PIK3C3,ERAS,CHRNA7,CHRFAM7A,INSR,RAB20,DDAH1,RASGRP3,RASGRP1,GNG7,RAP2C,TNIK,LYN,STMN3,PTGER4,RALBP1,SLA2,RAB4A,TIFA,CYP26A1, IFI16,DAPK1,NRIP1,RAD1,MAP4K4,PRKCQ,SS18,DOK3,DOK4,PIAS2, RAB7B,FGFR3,ADORA2B,GNAI1,C5,DUSP10,PML,NFKBIA,KIT,RAB40B,CD74,ORO2A,IGF1R,DUSP9, RASGRP2
Hemopoietic or lymphoid organ development	21	PTPRC, TWSG1, LYN, FLT3, RELB, PML, KITLG, SOX4, CDK6, MALT1, KIT, ITGA4, IFI16, ZBTB16, CD74, TAL1, BCL2, CASP8, JAK2, RUNX1, HDAC9
Positive regulation of cell differentiation	19	PTPRC, LYN, PLXNB2, CLU, DLL3, PAX6, SMAD3, KITLG, KIT, GLI3, CD74, EPHB2, TAL1, BCL2, JAK2, IL2RG, CA2, RUNX1, ZFHX3
Hemopoietic progenitor cell differentiation	4	FLT3, BCL2, SOX4, KIT
Cell differentiation	87	FLT3,HSPG2,SMAD3,MALT1,ITGA4,GAS1,HGF,AFG3L2,ZSCAN2,NOTCH3,DLX2,DLX1,ITGA6,JAK2,AACS,HDAC9,PBX3ALS2,CAST,S100A4,MEF2C,KITLG,ZBTB16,SPATA20,ZAK,ATL1,TSNAX,PAX6,CBFA2T3,PTEN,GLI3,BTK,GATA2,BAG1,CD44,CASP8,ERAP1,ANGPT1,BHLHE41,INSR,INA,LYN,DIAPH2,CLU,PML,SOX4,EGFL7, STMN3, PLXNB2, RELB, DLL3, CYP26A1, CDK6, IFI16, PRDM8, PRKCQ, MIB1, SDC1,NAV1, ZMIZ1, ZFPM2, IFT81, EFNA5, BIN1, EID1, TWSG1, RTN4RL1, KIT,TIMP2,TTC3,CD74,SCARF1,EPHB2,ANGPTL6,TAL1,IGF1R,GNPTAB,RASGRP1,BCL2,PVRL2,PPP3CA,GPNMB,RUNX1, NFATC1, TXNIP, PTPRC, CCM2

Appendix Table S7. Sequences of primers used in this study

Names		Sequence from 5' to 3'	Product size (nt)
**Transactivation Assays**			
THAP10-P1	sense	CGGGGTACCGGCTCCTGATTTCAGAAATGTGT	1982
THAP10-P2	sense	CGGGGTACCTTCAGCTCAGGAGCTCGAGA	1689
THAP10-P3	sense	CGGGGTACCGGCAGAGGTTGCAGTGAGCCA	1541
THAP10-P4	sense	CGGGGTACCCTGGTAAATGATCTTGTTCTGAGGA	1289
THAP10-P5	sense	CGGGGTACCTTTCACAGCAGACCAGCTCCA	741
THAP10-P6	sense	CGGGGTACCAGGCAACCTGCTCAGGGTT	363
THAP10-P7	sense	CGGGGTACCTGGCCACACTGGTTGCTATAAC	165
THAP10-P8	sense	CGGGGTACCGCGAAATTTGTATCCCTCTGCT	91
Antisense for THAP10		CGGGGTACCAATGGGAGGGCTCTTTGATATC	
MIR383-P1	sense	CGGGGTACCATTTCTTTCTTCAGAGATTTGAAGT	1491
MIR383-P2	sense	CGGGGTACCATTTACTTTGATGTTGGCTACTGGT	850
MIR383-P3	sense	CGGGGTACCTCTGTAATATGCATTTACATGAAGT	456
MIR383-P4	sense	CGGGGTACCTCATGATTCTGTGAGAGTCACT	96
Antisense for MIR383		CCGCTCGAGAGGTGACGTGGAGTTTCTGG	
**siRNA**			
si-AML1-ETO	siAGF1-a	CCUCGAAAUCGUACUGAAAG	
	siAGF1-b	UUGGAGCUUAGCAUGACUCU	
Negative control	sense	UUCUCCGAACGUGUCACGUUT	
	antisense	ACGUGACACGUUCGGAGAATT	
Anti-miR-383		AGCCACAAUCACCUUCUGAUCU	
Mimics control		UUGUACUACACAAAAGUACUG	
**ChIP Assay**			
Oligo for THAP10 target	sense	ATTTCCGGTCTGGACCTCTC	163
	antisense	AGCCTCTTCCCCAGCTTATC	
Oligo for THAP10 Off-target	sense	GCCCTCCCAATTACCATTTT	197
	antisense	CTCCCAAAGTGCTGGGATTA	
Oligo for MIR383 target	sense	AGCCCAACATTTACAAGTCA	184
	antisense	TAACGACATCAGTTTCTA	
Oligo for MIR383 Off-target	sense	ATGGACACTGTTGAGGGTA	207
	antisense	TCCCCAGCCGGCATCTCCAT	
GAPDH	sense	TACTAGCGGTTTTACGGGCG	209
	antisense	TCGAACAGGAGGAGCAGAGAGCGA	
**Bisulfite modification and genomic sequencing**			
Oligo for THAP10	sense	GTGGGAAGAAAGAGAAAGTAG	359
	antisense	AACTTATCCTCCTCCCAATAC	
**Plasmid**			
THAP10-FLAG	sense	CCCAAGCTTATGCCGGCCCGTTGTGTGGCCGCCCACT	771
	antisense	CCGCTCGAGACATGTTTCTTCTTTCACCTGTACA	
tr-THAP10-GFP	sense	CCCAAGCTTATGCGGGTGCCCGCCCCGGCA	504
	antisense	CCGCTCGAGACATGTTTCTTCTTTCACCT	
THAP10-GFP	sense	CCCAAGCTTATGCCGGCCCGTTGTGT	768
	antisense	CCGCTCGAGACATGTTTCTTCTTTCACCT	
THAP10-3'UTR	sense	GCTCTAGACTCAACATCAAATGCTCTGATGTGC	1114
	antisense	AACTGCAGGATGTATTATTTAAACCTTCTAGG	

THAP10-3'UTR-miR-383 binding site mutant	sense<br>antisense	GCATACTATAACTGTCAACATCCATTGAGA<br>TCTCAATGGATGTTGACAGTTATAGTATGC	1114
miR-383	sense<br>antisense	CGCGGATCCCAGTTCTTTAAAGGACAAAGC<br>CCGGAATTCACAGGTTAAACATGGCAC	269
**qPCR**			
FGFR3	sense<br>antisense	ACTGTCTGGGTCAAGGATGG<br>TGTGTCCACACCTGTGTCCT	189
PDGF	sense<br>antisense	GCTTTGGCTTTGGCTATCAG<br>CACTCTGTCTGCCCTTCTCC	216
PML	sense<br>antisense	CTGAGGTGATGCTGGCTACA<br>AAGCAAGCTTTGGAAGGTCA	203
ABI1	sense<br>antisense	AAGTGGCTAAAAGCCAAGCA<br>AAGCCTAGCAGGACTGGTCA	201
KIT	sense<br>antisense	TCATGGTCGGATCACAAAGA<br>AGGGGCTGCTTCCTAAAGAG	196
PTEN	sense<br>antisense	ACCAGGACCAGAGGAAACCT<br>GCTAGCCTCTGGATTTGACG	194
PDGFC	sense<br>antisense	TGAGTTTTCGCCTCTGTCCT<br>AGCCCTCTTCTGTGTCTCCA	186
FLT3	sense<br>antisense	ACGTGTGCTTTTACCCCAAG<br>CTTCTGACTGGCCCTGAGTC	226
HGF	sense<br>antisense	GGGCTGAAAAGATTGGATCA<br>TTGTATTGGTGGGTGCTTCA	208
JAK2	sense<br>antisense	GAGCCTATCGGCATGGAATA<br>ACTGCCATCCCAAGACATTC	215
ANGPT1	sense<br>antisense	GAAGGGAACCGAGCCTATTC<br>GGGCACATTTGCACATACAG	212
STAP2	sense<br>antisense	CCTGAAGCCAAAGAAGTTGC<br>TTGGGCTCTGGAAGAGAAGA	189
BCL2	sense<br>antisense	GGATGCCTTTGTGGAACTGT<br>AGCCTGCAGCTTTGTTTCAT	198
SOX4	sense<br>antisense	CCAGCAAGAAGGCGAGTTAG<br>CGGAGCCTTCTGTCTTCATC	206
PTPRG	sense<br>antisense	GCTGTGGATGTTTTCCAGGT<br>CCCTTTCAGGATTCCAGTCA	209
ABL1	sense<br>antisense	CTCCATTATCCAGCCCCAAA<br>CCCAGCTTGTGCTTCATGGT	189

Appendix Figure S1

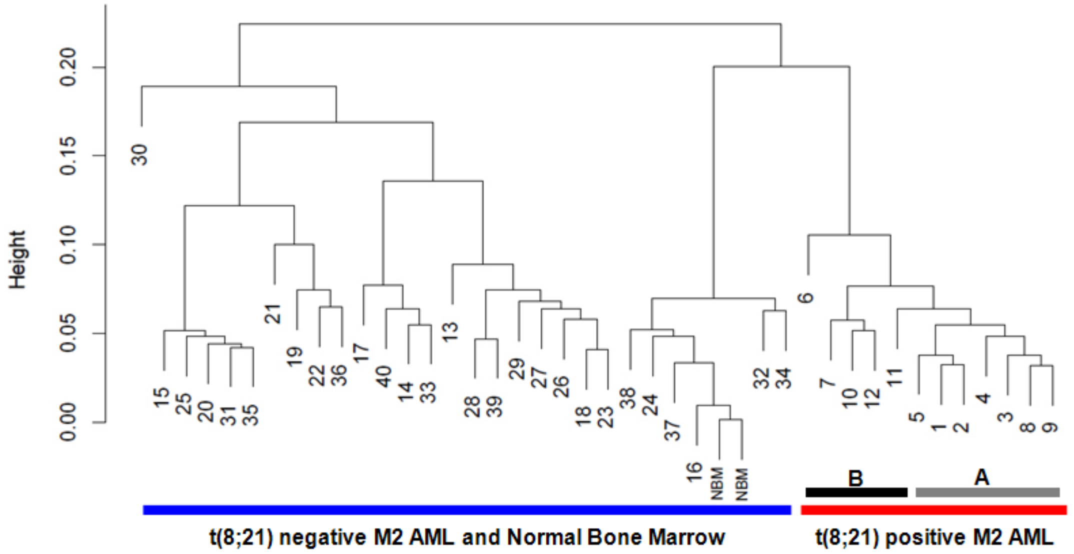

Appendix Figure S1. A dendrogram illustrating hierarchical clustering in blasts of 12 AML1-ETO^{+} (red) and 28 AML1-ETO^{-} FAB M2 AML patients (blue), as well as 2 normal bone marrow blasts (NBM, blue). Grey, subcluster A, in which 5/7 with Y chromosome deletion (-Y); black, subcluster B.

Appendix Figure S2

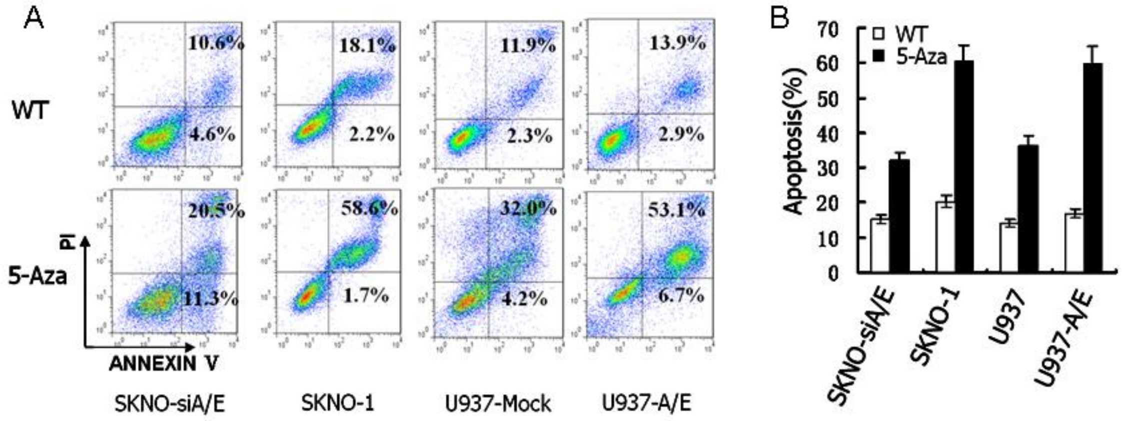

Appendix Figure S2. AML1-ETO^{+} cells are more sensitive to the demethylation reagent 5-Aza.

A Representative flow cytometric analysis of AML1-ETO^{+} cell lines (SKNO-1 and U937-A/E) and AML1-ETO^{-} cell lines (SKNO-siA/E and U937-Mock), after treated with 5-Aza (2.5 μM) for 40 hrs.

B Histograms for three independent evaluations (mean ± SD).

Appendix Figure S3

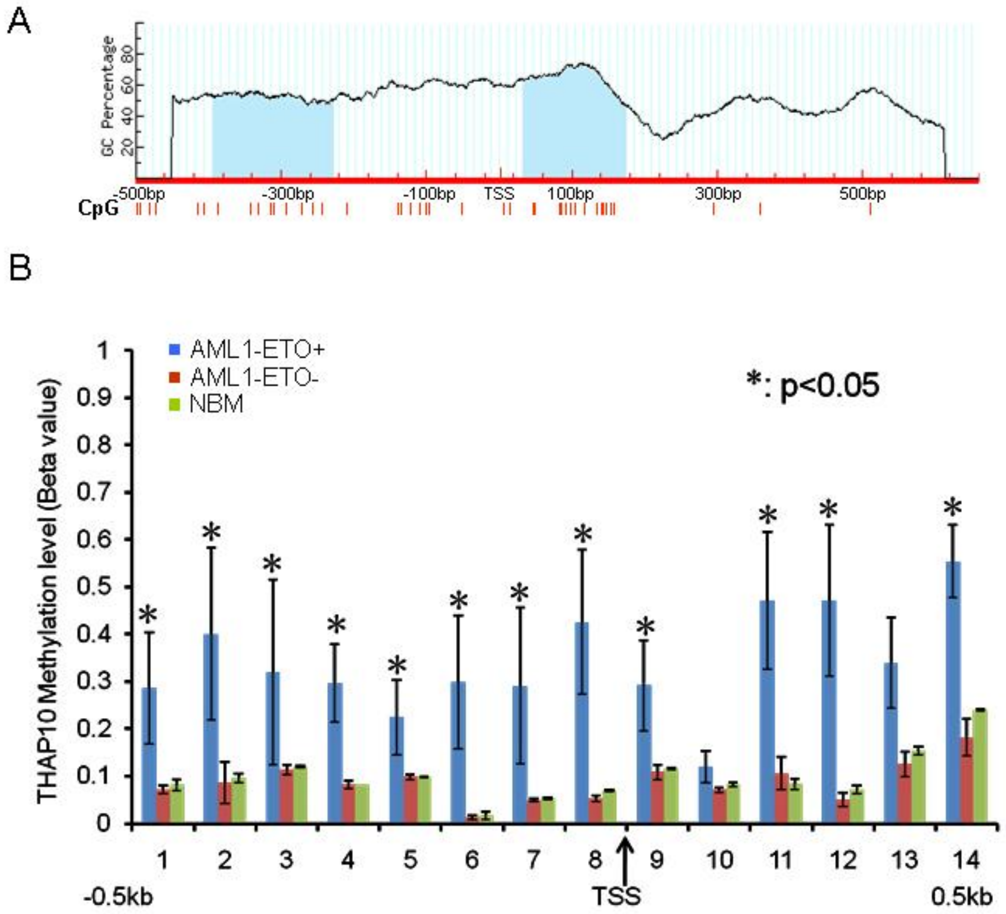

Appendix Figure S3. The methylation levels in the promoter region of *THAP10*.

A A schematic diagram of the CpG islands around the *THAP10* gene. Numbers indicate the nucleotides relative to *THAP10* TSS (transcriptional start site, +1). Vertical lines indicate the CpG dinucleotides.

B The average methylation levels of each probe in the promoter region in 12 AML1-ETO^{+} (blue), 28AML1-ETO^{-} (red), and normal samples (green). The number of probes are ordered by the coordinate relative to TSS, from -0.5 kb to 0.5 kb. Arrow indicates the position of TSS. *P< 0.05.

Appendix Figure S4

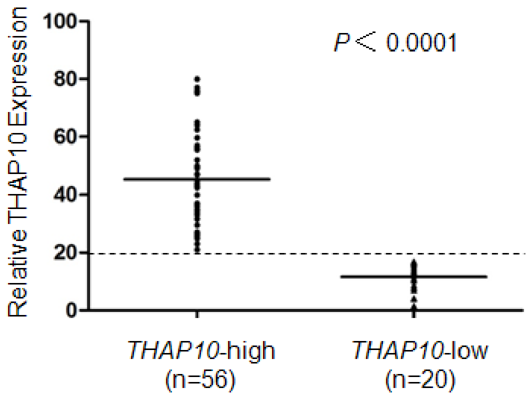

Appendix Figure S4. Patients with t(8;21) AML were grouped into quartiles according to *THAP10* levels and divided into *THAP10*-high and *THAP10*-low patients.

As shown by qRT-PCR, *THAP10* levels were significantly higher in *THAP10*-high (n = 56) group than *THAP*-low (n=20) group. A dashed line shows the cut-off value (2×10^1) of *THAP10* level.

Appendix Figure S5

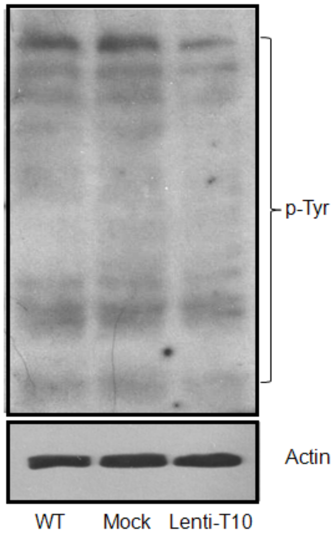

Appendix Figure S5. Total tyrosine phosphorylation in Kasumi-1 cells transduced with lentivirus-*THAP10*.

Kasumi-1 cells transduced with lentivirus-*THAP10* (Lenti-T10) or lentivirus vector (Mock) were collected, and western blots were performed to detected the total tyrosine phosphorylation levels. Antibodies p-Tyr (1:200, sc-51688, Santa Cruz Biotechnology) were used to detected p-Tyr levels in cell lines indicated. β-Actin was used as loading control.

Appendix References

Reference

Bennett JM, Catovsky D, Daniel MT, Flandrin G, Galton DA, Gralnick HR, Sultan C (1985) Proposed revised criteria for the classification of acute myeloid leukemia. A report of the French-American-British Cooperative Group. Ann Intern Med 103**:** 620-625
