# Supplementary material for: A novel epigenetic AML1‐ETO/THAP10/miR‐383 mini‐circuitry contributes to t(8;21) leukaemogenesis
Source: EMBO Mol Med. 2017 May 24;9(7):933–49. doi: 10.15252/emmm.201607180 (PMC5577530; doi:10.15252/emmm.201607180)
Supplement: Supplementary file 2 — Expanded View Figures PDF [file EMMM-9-933-s002.pdf]

Expanded View Figures

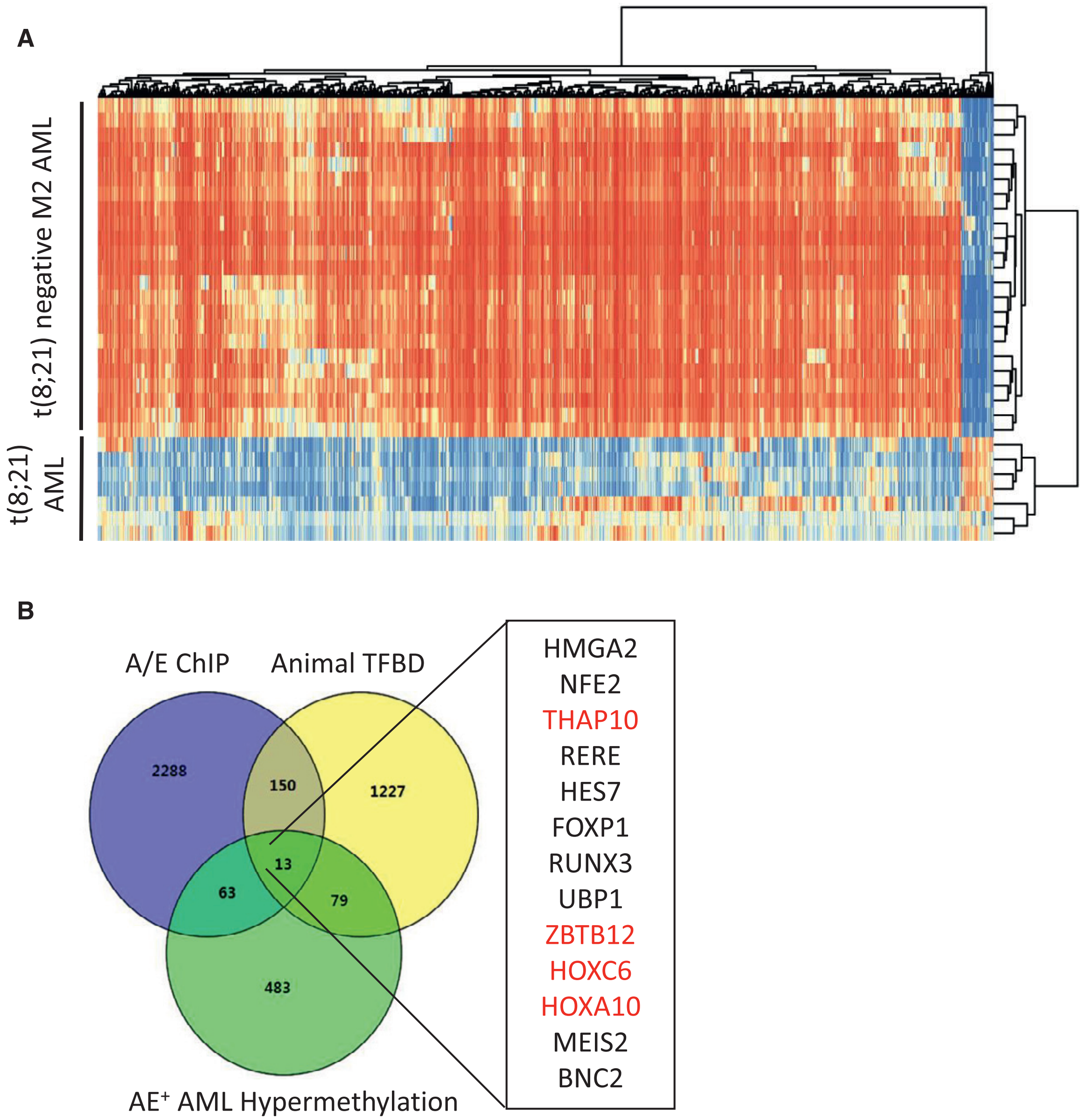

Figure EV1. TCGA methylome database reanalysis showed that AML1-ETO^{+} AML displays a similar unique genome-wide methylation signature compared to our data.

- A Overview of two-way (genes against samples) hierarchical clustering of AML1-ETO^{+} (n = 7) and AML1-ETO^{-} (n = 37) M2 AML blasts using the genes that vary the most among samples. Representative heatmap is shown to identify the aberrant DNA methylation signatures of specific genes in AML. Each column of the heatmap represents one gene, and each row represents one AML patient.
- B Venn diagrams for systematic survey of the publicly available database to identify the 13 genes in t(8;21) AML. Four out of 13 genes overlapped with our distinct signature in t(8;21) AML and are shown in red, including *THAP10*.

Source data are available online for this figure.

 © 2017 The Authors

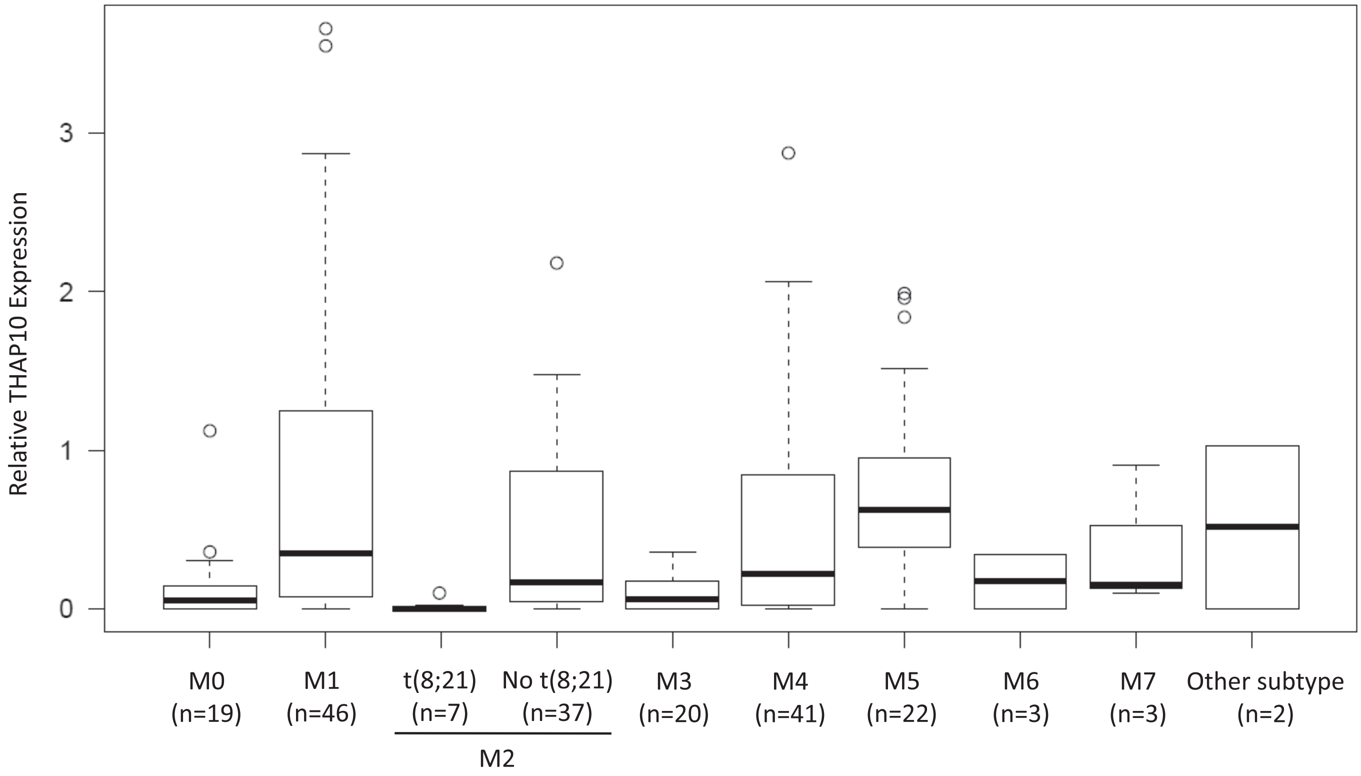

Figure EV2. *THAP10* mRNA levels in AML subtypes from TCGA databases.
Boxplot analysis showed that *THAP10* mRNA levels were the lowest in t(8;21)$^+$ AML. AML subtypes were classified according to the FAB classification. Data are represented as a boxplot, which shows the median value of mRNA levels (line in the middle of the box), the first and third quartiles (lower and upper limits of each box, respectively). THAP10 expression is derived and normalized from RPKM (Reads Per Kilobase per Million mapped reads) of RNA-Seq data for 200 AML samples. Whiskers display the highest and lowest data values.

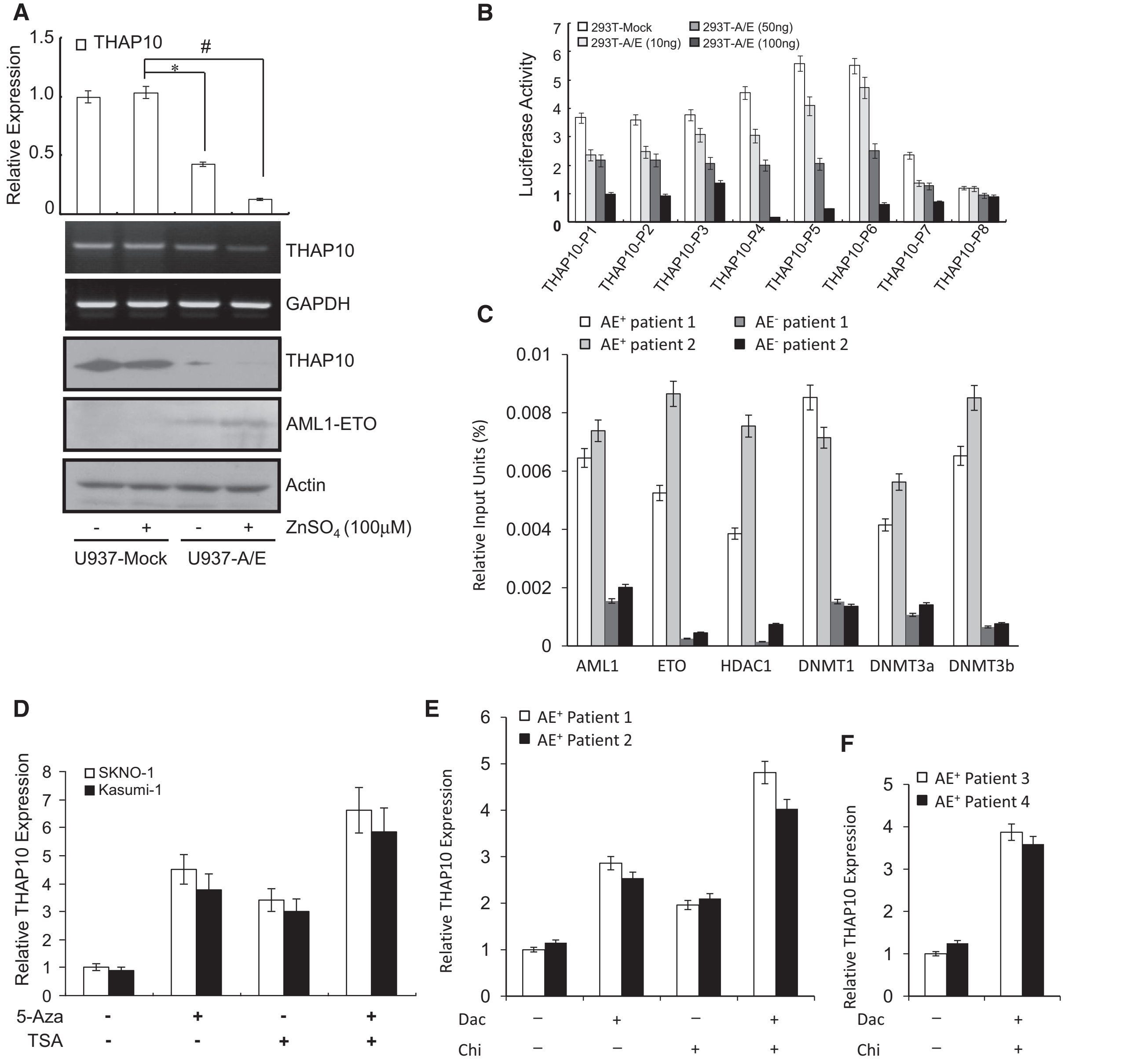

Figure EV3. Epigenetically down-regulated *THAP10* can be reactivated by DNMT inhibitors and HDAC inhibitors.

A Upper panel: Relative quantification of *THAP10* levels in U937 (Mock and A/E-HA) cells. The results represent the average of three independent evaluations ± SD (*P = 0.038, #P = 0.008, two-sided Student's *t*-test was used for the comparisons). Middle panels: RNA levels of *THAP10* analysed by agarose electrophoresis. *GAPDH* was used as a loading control. Lower panels: Protein levels of THAP10 and AML1-ETO monitored by immunoblot analysis. β-actin was used as a loading control. Treatment for 16 h with 100 μM Zn^{2+} was employed to induce the expression of *AML1-ETO*.

B Human 293T cells were transiently co-transfected for 48 h with luciferase reporter containing wild-type sequences of the *THAP10* promoter regions, together with increasing amounts (10, 50 and 100 ng) of pcDNA3.0 with *AML1-ETO* cDNA or without (293T-Mock). Data represent the mean of three independent evaluations ± SD for luciferase activity relative to the empty vector (pGL3-LUC) control. pRL-TK was used as an internal control.

C Chromatin immunoprecipitation (ChIP) using the indicated antibodies or IgG in AML blasts, after which qRT–PCR was performed to evaluate the specificity of protein binding. Triplicate values are defined as mean ± SD.

D SKNO-1 and Kasumi-1 cells were treated with the DNMT inhibitor 5-azacytidine (5-Aza, 2.5 μM) +/− the HDAC inhibitor TSA (3.0 μM) for 40 h, after which RT–PCR was performed to detect the mRNA levels of *THAP10*. Data represent the normalized values (mean ± SD) of relative *THAP10* expression from three independent experiments. Normalized *THAP10* level in SKNO-1 cells was arbitrarily set to 1.

E AML-ETO+ patient blasts were treated with the DNMT inhibitor decitabine (Dac, 2.5 μM) +/− the HDAC inhibitor chidamide (Chi, 3.0 μM) for 40 h, after which RT–PCR was performed to detect the mRNA levels of THAP10. Data represent the normalized values (mean ± SD) of relative *THAP10* expression from three independent experiments. Normalized *THAP10* level in SKNO-1 cells was arbitrarily set to 1.

F In primary blasts from clinical trial (NCT02886559) patients, qRT–PCR detected the *THAP10* levels right after completion of the first therapeutic program. Normalized *THAP10* level in patient blasts was arbitrarily set to 1. Triplicate values are defined as mean ± SD.

 © 2017 The Authors

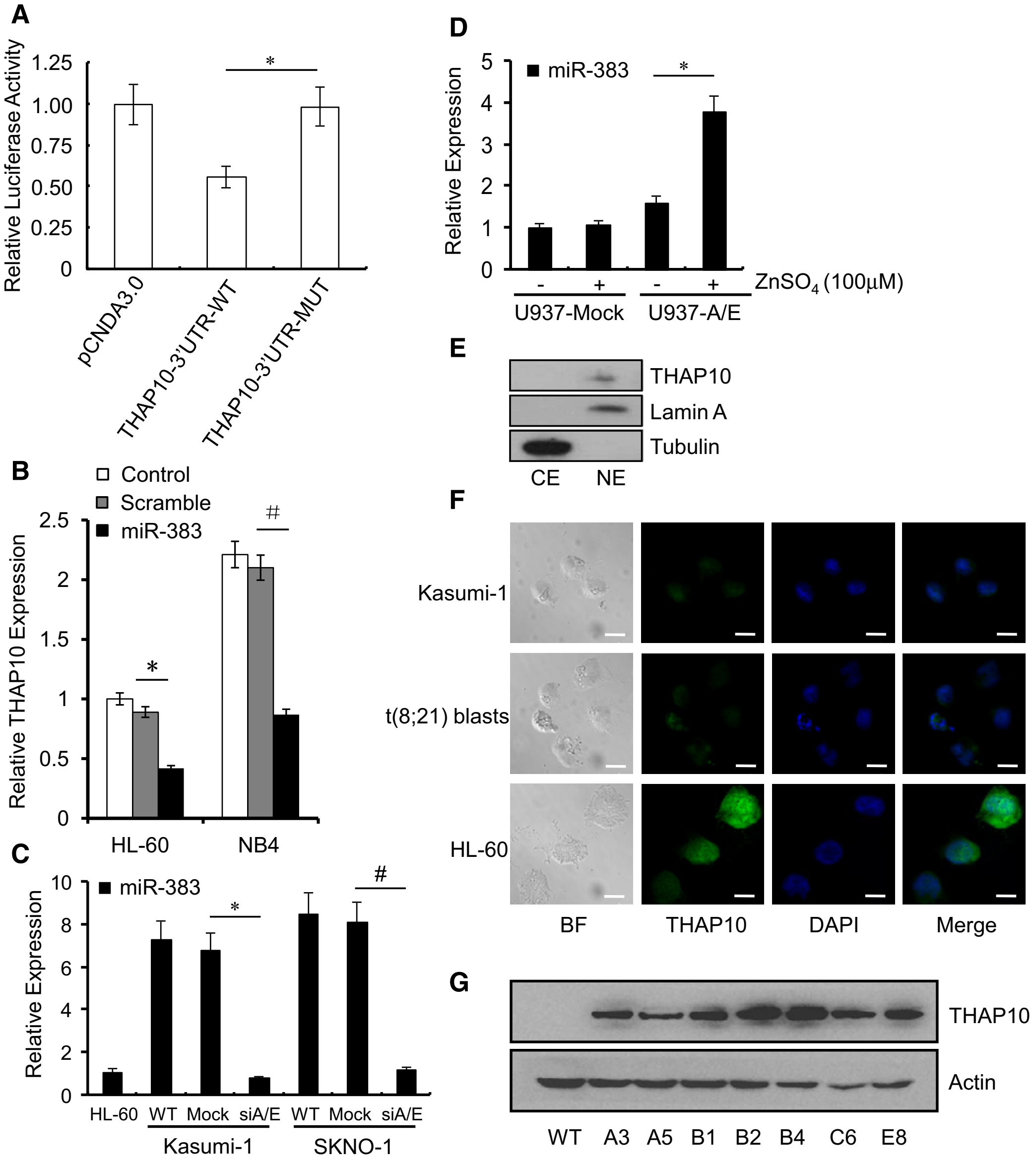

Figure EV4. *miR-383* levels in indicated cell lines and the localization of THAP10.

A HEK293T cells were co-transfected for 48 h with pCDNA3.0 control and *miR-383* expressing plasmids, together with a modified luciferase pGL-3 control vector containing *THAP10* 3′-UTRs or a predicted *miR-383* binding site mutant as indicated, after which luciferase activity assays were performed. Data represent the normalized values (mean ± SD) of relative luciferase activity from three independent experiments (*P = 0.009, two-sided Student's t-test was used for the comparison). Normalized luciferase activity in the absence of the *miR-383* expression vector was set to 1.

B Relative quantification of *THAP10* levels in HL-60 and NB4 leukaemic cells after transfection with synthetic *miR-383* or scramble for 48 h. The results represent the mean of three independent evaluations ± SD (*P = 0.0001, #P = 0.0001, two-sided Student's t-test was used for the comparisons).

C Relative quantification of *miR-383* expression in the indicated cells. The results represent the mean of three independent evaluations ± SD (*P = 0.001, #P = 0.0015, two-sided Student's t-test was used for the comparisons).

D Relative quantification of *miR-383* levels in U937 (mock and A/E-HA) cells. The results represent the mean of three independent evaluations ± SD (*P = 0.004, two-sided Student's t-test was used for the comparison).

E Endogenous expression of THAP10 in nuclei. Cytoplasmic (CE) and nuclear extracts (NE) were isolated from Kasumi-1 cells transduced with lentivirus vector expressing *THAP10*, after which Western blot analysis was performed using polyclonal anti-THAP10 antibody. Tubulin and lamin A were used as loading controls for the cytoplasm and nuclei, respectively.

F Immunofluorescence microscopy shows the endogenous THAP10 localization in Kasumi-1, HL-60 and t(8;21) AML blasts. BF, bright field. Scale bar, 10 μm.

G Ectopic expression of *THAP10* in Kasumi-1 cells. After transduction with lentivirus vector expressing *THAP10*, multiple stable clones indicated with capital letters and numbers were selected after incubation with puromycin for 30 days. Western blot analysis using anti-THAP10 antibody was performed to screen and monitor the protein levels of THAP10. β-actin was used as a loading control. WT, wild type.

Source data are available online for this figure.

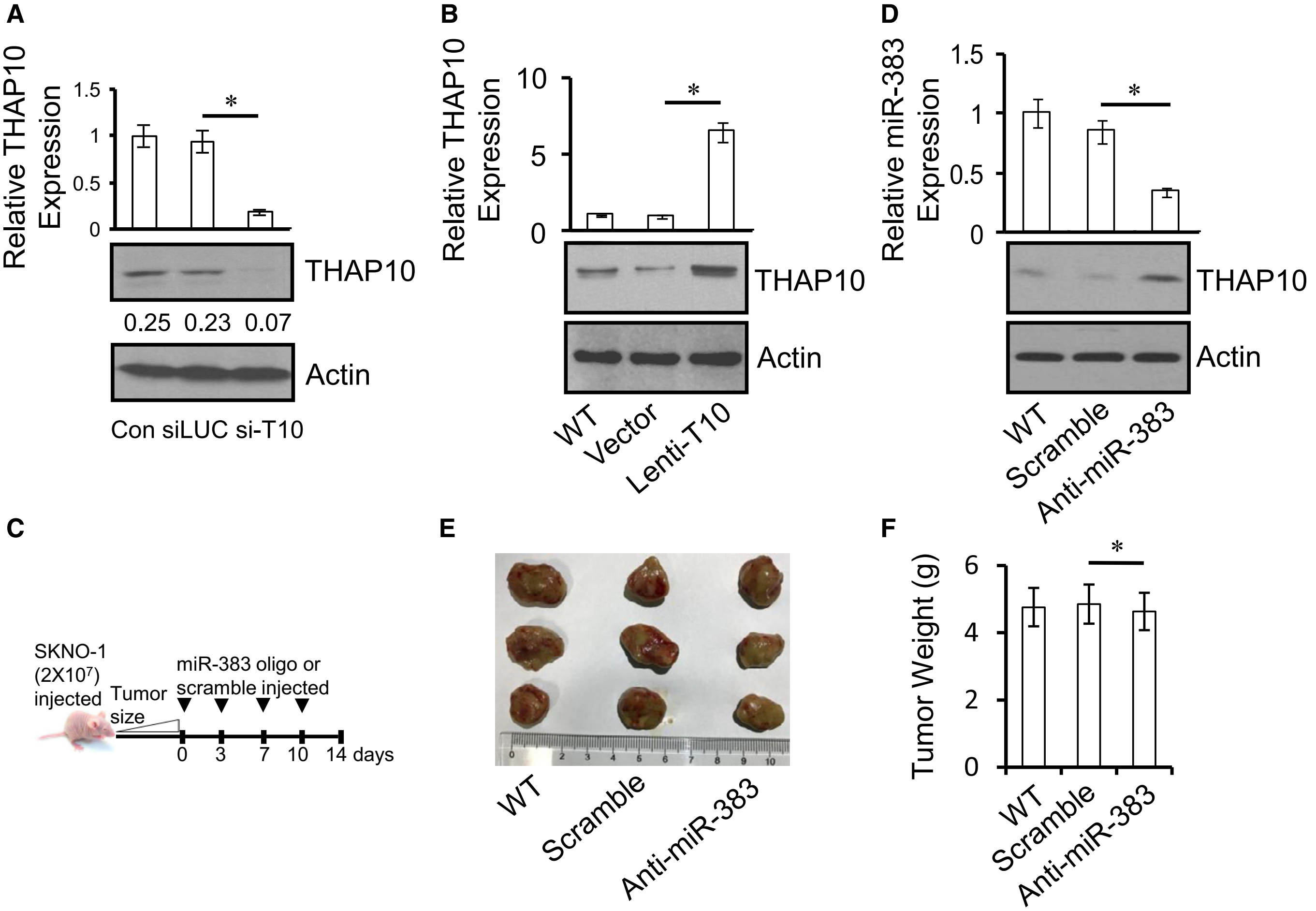

Figure EV5. Levels of *THAP10* and *miR-383* in cell lines or tumours and the mouse model.

A Upper panel: Relative quantification of *THAP10* mRNA levels in HL-60 cells (wild type, siLUC and si-T10). The results represent the mean of three independent evaluations ± SD (*P = 0.002, two-sided Student's t-test was used for the comparison). Lower panels: Immunoblot analysis of THAP10 protein levels using an anti-THAP10 antibody. β-actin was used for a loading control. Values indicate the ratios between THAP10 and actin.

B Expression of *THAP10* in mouse Kasumi-1 cell xenograft models (wild type, lenti-empty vector and lenti-THAP10). Upper panel: Relative quantification of *THAP10* mRNA levels. The results represent the mean of three independent evaluations ± SD (*P = 0.0013, two-sided Student's t-test was used for the comparison). Lower panels: Protein levels of THAP10 by immunoblot analysis for THAP10 with an anti-THAP10 antibody in xenografts indicated. β-actin was used as a loading control.

C Diagram illustrating the workflow of the mouse study for testing the function of *miR-383*.

D Effects of *miR-383* on *THAP10* expression in mouse Kasumi-1 cell xenograft models (wild type, scramble and anti-*miR-383*). Upper panel: Relative quantification of *miR-383* levels. The results represent the mean of three independent evaluations ± SD (*P = 0.0029, two-sided Student's t-test was used for the comparison). Lower panels: Protein levels of THAP10 monitored by immunoblot analysis. β-actin was used as a loading control.

E Representative photographs of mouse xenografts in the scramble and synthetic anti-*miR-383* treated AML1-ETO^{-} HL-60 cells, captured at the end of the experiment (day 14).

F Triplicate values are defined as mean ± SD of tumour weight at the end of the experiment (WT, wild type; *P = 0.700, two-sided Student's t-test was used for the comparison).

Source data are available online for this figure.

 © 2017 The Authors
