# Supplementary material for: A novel epigenetic AML1‐ETO/THAP10/miR‐383 mini‐circuitry contributes to t(8;21) leukaemogenesis
Source: EMBO Mol Med. 2017 May 24;9(7):933–49. doi: 10.15252/emmm.201607180 (PMC5577530; doi:10.15252/emmm.201607180)

EMM-2016-07180_SourceDataForFigure EV 5A,5B and 5C

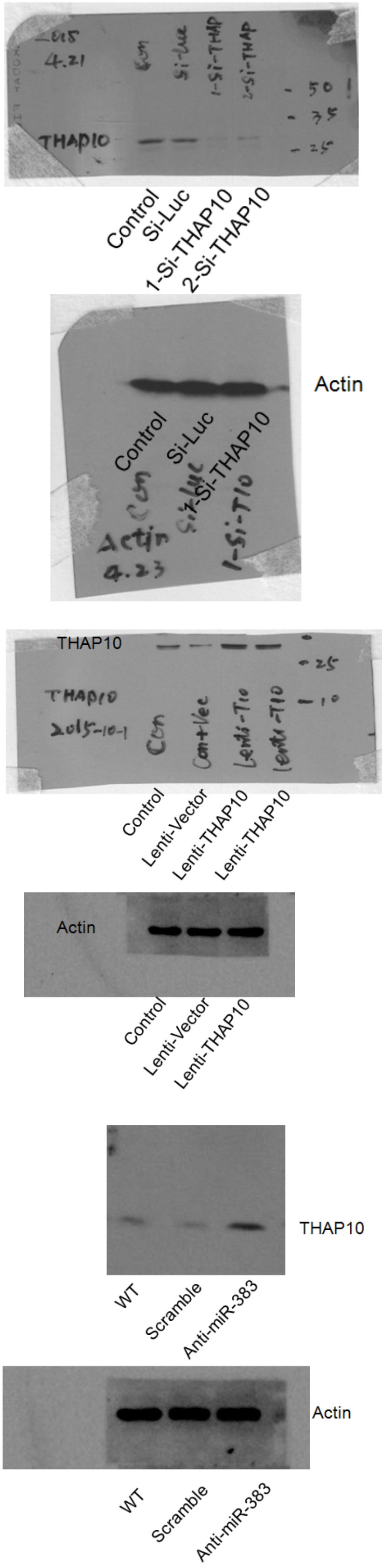

Supplement: Supplementary file 3 — Source Data for Expanded View [file EMMM-9-933-s008.zip › EMM_07180_EV_Source_data/EMM_07180_FigEV5ABC_SD.pdf]
