# Supplementary figures and images for: A novel epigenetic AML1‐ETO/THAP10/miR‐383 mini‐circuitry contributes to t(8;21) leukaemogenesis
Source: EMBO Mol Med. 2017 May 24;9(7):933–49. doi: 10.15252/emmm.201607180 (PMC5577530; doi:10.15252/emmm.201607180)

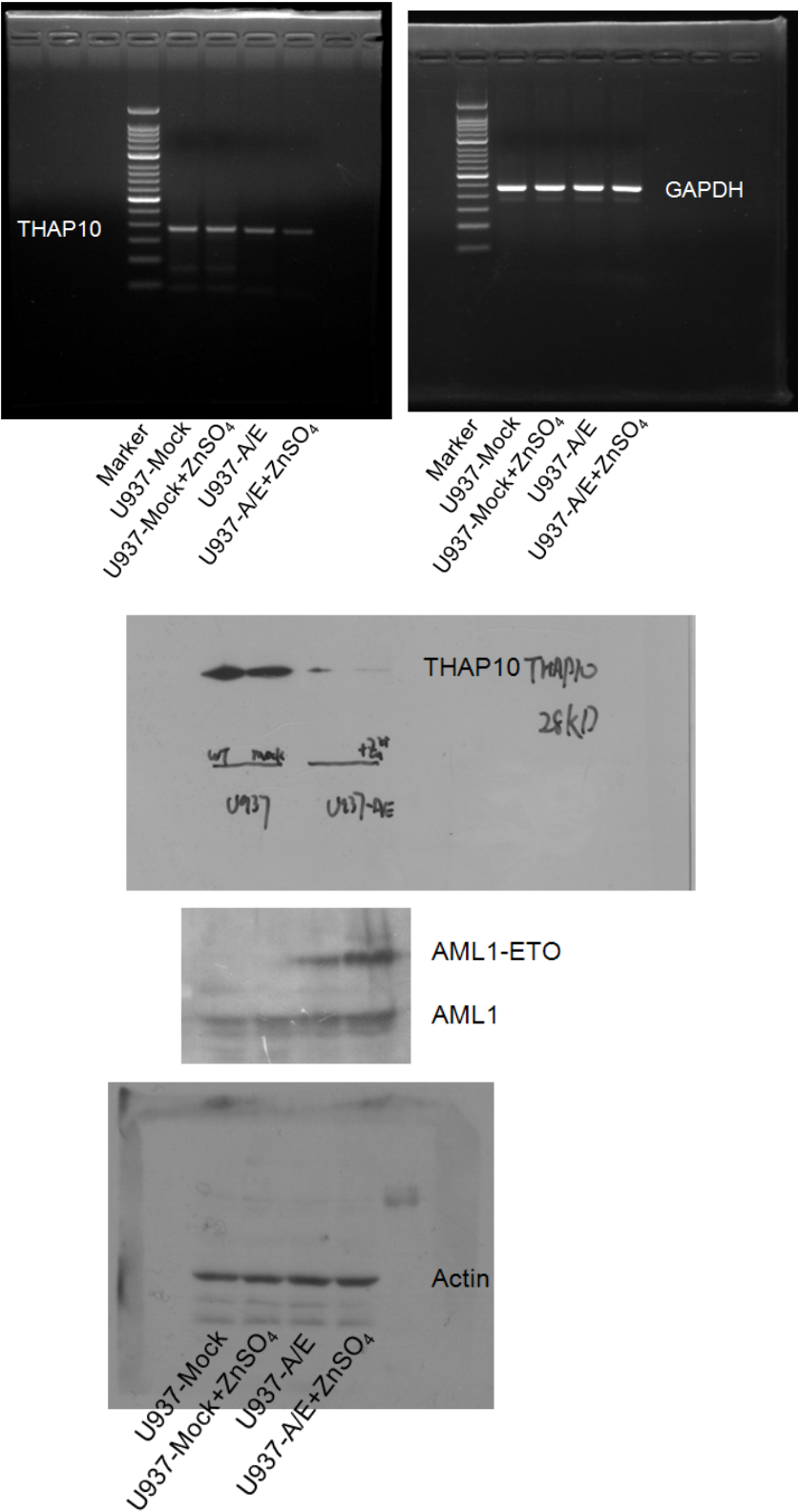

Supplement: Supplementary file 3 — Source Data for Expanded View [file EMMM-9-933-s008.zip › EMM_07180_EV_Source_data/EMM_07180_FigEV1A_SD.pdf]

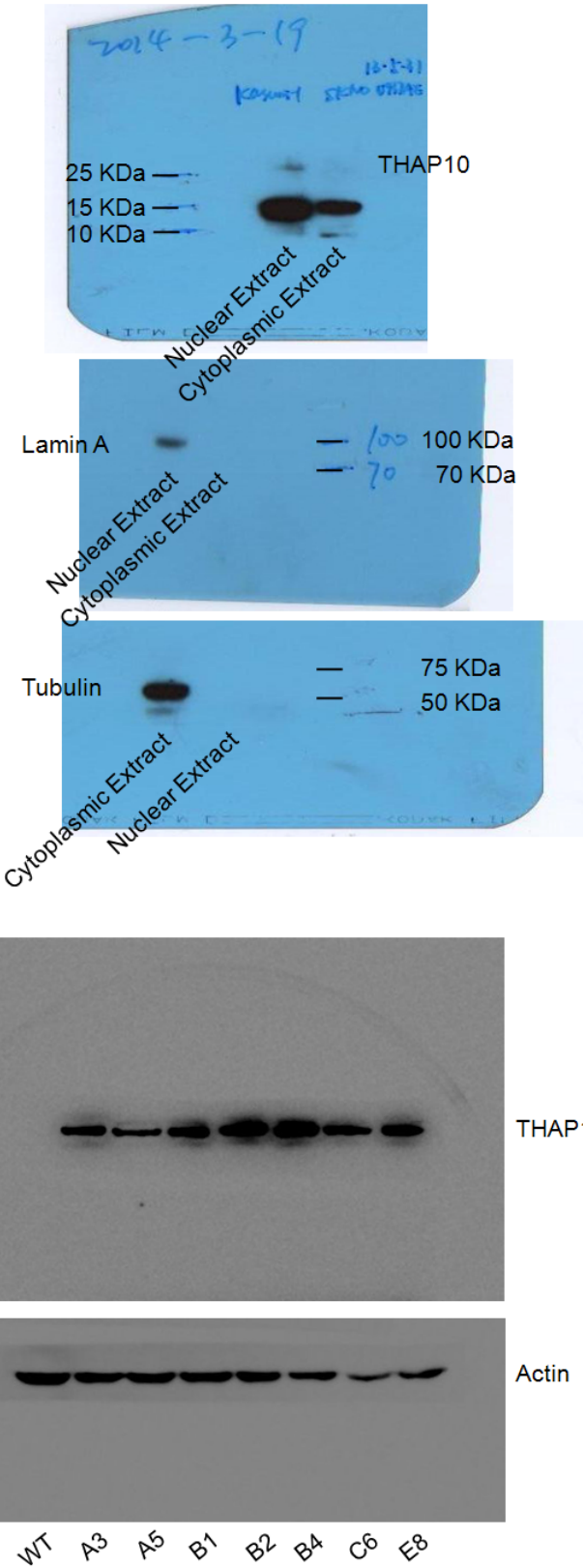

Supplement: Supplementary file 3 — Source Data for Expanded View [file EMMM-9-933-s008.zip › EMM_07180_EV_Source_data/EMM_07180_FigEV4EG_SD.pdf]

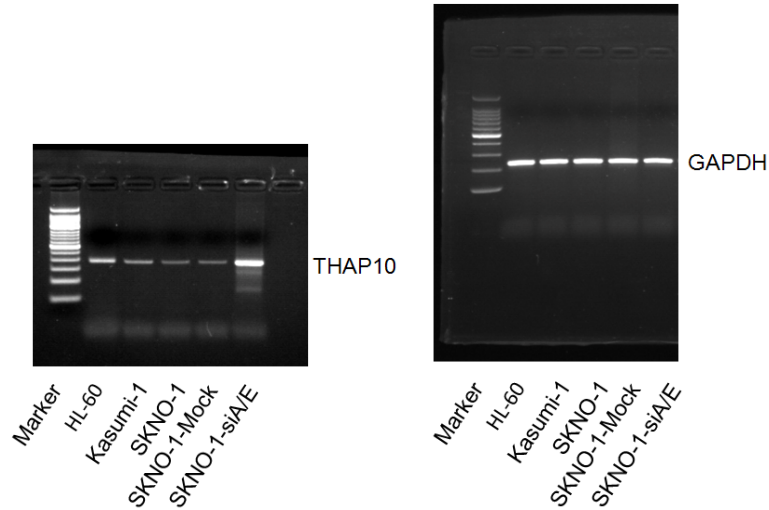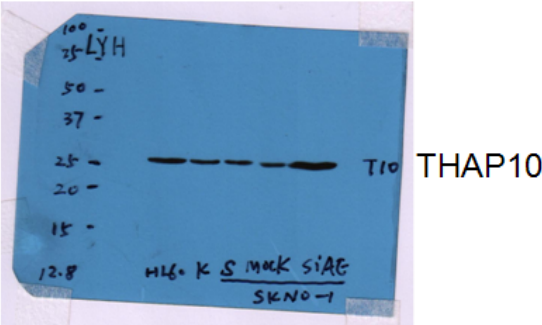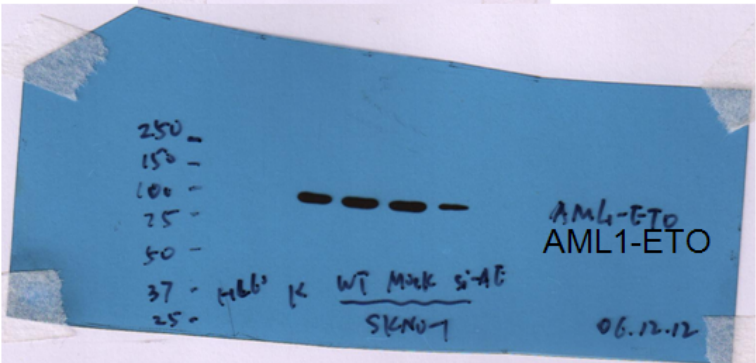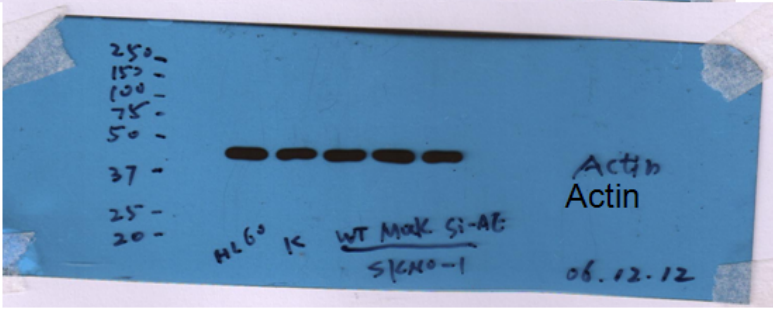

Supplement: Supplementary file 7 — Source Data for Figure 3 [file EMMM-9-933-s005.pdf]

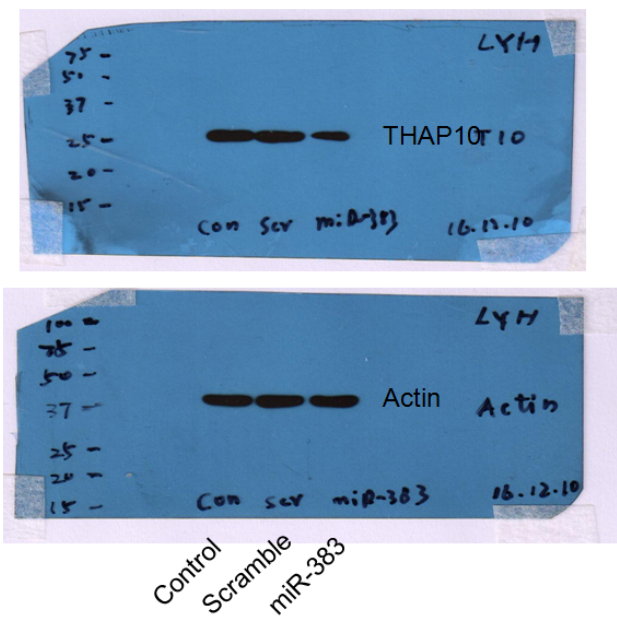

Supplement: Supplementary file 8 — Source Data for Figure 4 [file EMMM-9-933-s006.zip › EMM201607180_SourceDataForFigure_4B.pdf]

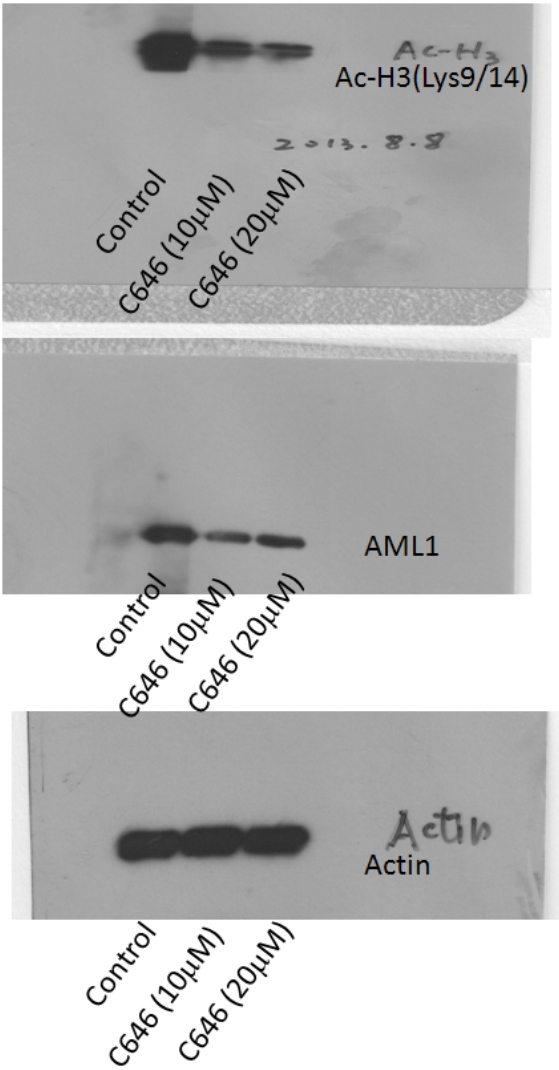

Supplement: Supplementary file 9 — Source Data for Figure 5 [file EMMM-9-933-s007.pdf]
